# Ecstasy Exposure & Gender: Examining Components of Verbal Memory Functioning

**DOI:** 10.1371/journal.pone.0115645

**Published:** 2014-12-29

**Authors:** Jenessa S. Price, Paula Shear, Krista M. Lisdahl

**Affiliations:** 1 McLean Hospital – Harvard Medical School, Belmont, Massachusetts, United States of America; 2 Department of Psychology, University of Cincinnati, Cincinnati, Ohio, United States of America; 3 Department of Psychology, University of Wisconsin-Milwaukee, Milwaukee, Wisconsin, United States of America; The Scripps Research Institute, United States of America

## Abstract

**Objective:**

Studies have demonstrated verbal memory deficits associated with past year ecstasy use, although specific underlying components of these deficits are less understood. Further, prior research suggests potential gender differences in ecstasy-induced serotonergic changes. Therefore, the current study investigated whether gender moderated the relationship between ecstasy exposure and components of verbal memory after controlling for polydrug use and confounding variables.

**Method:**

Data were collected from 65 polydrug users with a wide range of ecstasy exposure (ages 18–35; 48 ecstasy and 17 marijuana users; 0–2310 ecstasy tablets). Participants completed a verbal learning and memory task, psychological questionnaires, and a drug use interview.

**Results:**

Increased past year ecstasy exposure predicted poorer short and long delayed free and cued recalls, retention, and recall discrimination. Male ecstasy users were more susceptible to dose-dependent deficits in retention than female users.

**Conclusion:**

Past year ecstasy consumption was associated with verbal memory retrieval, retention, and discrimination deficits in a dose-dependent manner in a sample of healthy young adult polydrug users. Male ecstasy users were at particular risk for deficits in retention following a long delay. Gender difference may be reflective of different patterns of polydrug use as well as increased hippocampal sensitivity. Future research examining neuronal correlates of verbal memory deficits in ecstasy users are needed.

## Introduction

Ecstasy (containing 3,4-methylenedioxymethamphetamine, or MDMA) is a popular recreational drug among adolescents and young adults Among 10^th^ and 12^th^ graders, the Monitoring the Future Study reports slightly increased rates of use in 2013 versus prior years, at 3.6% and 4%, respectively [1]. According to the Substance Abuse and Mental Health Services Administration (SAMSHA), the average age of first use for ecstasy is post-high school, at 19.4 years old [2]. In addition, individuals who use ecstasy are often polydrug users; in a sample of nearly 9000 people 18–29 years old, Keyes and colleagues [3] reported that nearly half of those currently using ecstasy were also using more than 3 other types of illicit drugs. As such, polydrug use in addition to cumulative ecstasy use (e.g., new-onset use and continued use thereafter) is an important consideration in the present study. Further, ecstasy consumption has been associated with serotonin (5-HT) release and reuptake and, to a lesser extent, dopamine and norepinephrine [4]–[6].

Of particular concern, animal models have suggested that ecstasy acts as selective serotonergic neurotoxin in several areas of the brain [7]–[10]. In humans, neuroimaging studies using positron emission tomography (PET) scans have shown reduced 5-HT transporter (SERT) densities as well as increased 5-HT 2A receptor binding, even among abstinent ecstasy users in several cortical regions, including the hippocampus, frontal, occipital, and temporal areas of the cortex [11]–[14]. Such findings suggest damage to the serotonergic system even with moderate levels of ecstasy use, such that SERT downregulates while 5-HT 2A upregulates [15]. In addition, a dose-dependent relationship between decreased SERT densities and increased lifetime ecstasy use has been reported [16]. It has been suggested that the hippocampus may be particularly sensitive to ecstasy-associated neurotoxicity [17]–[20]. Among rats given MDMA, serotonin neurotoxicity was greater in the hippocampus than in other brain regions surrounding the temporal lobe, such as the neocortex and parietal lobe [21]–[22]. Consistent with this finding in animals, adolescent ecstasy users demonstrated abnormal hippocampal activation to a verbal working memory fMRI task [23]. Additionally, Daumann and colleagues [24] examined brain activation during an fMRI task among adult abstinent ecstasy users (mean age  = 27 years) and noted abnormal activation patterns in frontal and temporal regions among those with histories of heavy use versus those with moderate use and nonusers.

Given the potential vulnerability of the hippocampus to ecstasy neurotoxicity, verbal memory functioning in ecstasy users is of great interest. Two meta-analyses suggest that, in comparison to ecstasy-naïve controls, ecstasy users demonstrate learning and memory deficits of magnitudes associated with medium (Cohen's d = −0.55) [25] to large (Cohen's d = −0.92) [26] effect sizes. Although these meta-analyses could have been influenced by the positive publication bias [27], it is noteworthy that with few exceptions [28]–[30], individual neuropsychological studies have suggested that ecstasy is most consistently associated with verbal learning and memory abnormalities, among former as well as recent, currently abstinent individuals [18]–[20], [31]–[37]. Studies that reported specific effects on *underlying components* of verbal memory have reported deficits in immediate memory [38]–[42], delayed memory [19], [39], [41]–[43], intrusions and repetitions [38], and recognition [41]–[42]. A recent study by Bosch and colleagues [44] examined brain glucose metabolism and verbal memory in a sample of emerging adult male ecstasy users and controls (mean age  = 23.8 years). Among users, results indicated correlations between prefrontal and parietal hypometabolism and learning and recall deficits, and between mediotemporal hypometabolism and recognition deficits. With respect to performance on a prose recall task, there has been an inability to replicate the deficits found among ecstasy users in Morgan et al.'s [45] sample [34], [46]–[47].

Consistent with differences in the developmental trajectory of specific brain regions between men and women, particularly the hippocampus [48]–[52], it may be possible that gender moderates the effects of ecstasy on memory. Furthermore, gender differences in underlying 5HT functioning have been reported. For example, low cerebral spinal fluid 5HIAA (5-hydroxyindoleacetic acid; a serotonin metabolite considered as a measurement of serotonin turnover) has been associated with increased risk for serotonin-related diseases [53]–[54]. Some studies have reported that levels of the serotonin metabolite differ as a function of gender and genetic interactions, with one mechanism being the effect of estrogen on signal transduction and gene expression affecting serotonin functioning [55]–[58]. Allott and Redman [59] reviewed the role of gender in ecstasy effects and concluded that female users appear more vulnerable to variability in 5-HT functioning as well as to short term dose-dependent psychological and physical symptoms, while male users tend to show increased acute physiological effects. However, not all studies examined differences in dose between genders, and few neurocognitive studies have examined potential gender differences. Reneman et al. [60] reported greater reductions in SERT binding among female compared to male ecstasy users, after controlling for verbal intelligence, age, comorbid alcohol, nicotine and marijuana use, and premorbid Axis I psychiatric disorders. Of note, this study examined ecstasy dose between moderate- and heavy-using groups of men and women, finding that men within the heavy-using group reported increased exposure as compared to heavy-using women as measured by both number of tablets as well as dosage (tablets/weight ratios). In contrast, Bolla et al. [61] found that male ecstasy users demonstrated increased dose-dependent deficits on verbal and visual memory than female users.

The goal of the present study was to examine whether ecstasy use and gender interact in predicting components of verbal memory including immediate recall, proactive interference, short delayed recall, long delayed recall, discriminability, and retention after controlling for important demographic variables such as ethnicity, premorbid verbal ability, and family history of substance use disorders (SUD; which have been linked to neurocognitive abnormalities and increased risk for substance use disorders) [62]–[63]. Additionally, given that ecstasy users are typically polydrug users, the authors also sought to account for this issue through the inclusion of polydrug users with a full range of ecstasy use in the study sample as well as by controlling for polydrug use in the analyses.

## Methods

### Participants and Procedure

The Institutional Review Board at the University of Cincinnati approved this study and its parts. As part of a larger study (see Medina, et al. [20] for detailed methodological information), participants were recruited through community advertisements. We utilized a quota-bin sampling technique, balanced for gender, which allowed for sufficient representation of the expected range of ecstasy use (bin 1 =  no use, see below; bin 2 = 1–60 tablets; bin 3 = 61–200 tablets; bin 4 = >200 tablets). Due to heavy comorbid polydrug use among the ecstasy users, especially marijuana use, bin 1 included marijuana (MJ) users who had never used ecstasy. Bin 1 assessed a similar range of MJ use as demonstrated by ecstasy users (ranged from low to very heavy use). Therefore, the *combination of all bins* represented the full range of lifetime ecstasy use (from *none* to *heavy*), and data were analyzed *using a continuous variable reflecting lifetime or past year ecstasy exposure*. This analytic approach was chosen to closely examine the dose-dependent relationship across the full range of ecstasy usage while controlling for polydrug use (especially alcohol and marijuana).

Participants were recruited through community advertisements and screened by phone to assess eligibility. Eligibility criteria included ages 18–35, being fluent in English, having maintained abstinence from ecstasy and other substances for at least 7 days prior to the testing session, and reporting a lifetime ecstasy consumption level that fell into one of the open sampling bins. Axis I disorders (psychotic disorder, bipolar disorder, major depressive disorder, or anxiety disorder) were screened utilizing a modified SCID I/P interview based on DSM-IV criteria [64]. Interested participants who had positive responses to the screening questions were discussed in committee; if clear decisions could not be reached then they were re-contacted and administered the appropriate SCID I/P module by a trained interviewer. Individuals who met current diagnostic criteria for the aforementioned disorders (independent of their substance use) were excluded from the study. Participants were also excluded for a history of traumatic brain injury or other neurologic or major medical disorders, current psychiatric medications, or intellectual deficiency or learning disability.

Participants completed a brief background questionnaire, psychological questionnaires, and were administered a drug use interview and neuropsychological battery. Upon completion of the study, they were paid $35 and given informational pamphlets and drug and alcohol treatment referrals. A total of 48 (22 females, 26 males) ecstasy users and 17 MJ (8 males, 9 females) users completed the study.

### Screening Inventories and Questionnaires

#### Frequency/Quantity of Drug Use

A modified version [20], [51] of the *Time-Line Follow-Back* [65] technique was conducted, using memory cues like holidays and personal events to measure past year drug use. The *Time-Line Follow-Back* has demonstrated both high test-retest reliability as well as significant correlation with urine toxicology results [66]. In addition, a semi-structured interview was used to measure frequency of *lifetime* drug use, in which average weekly use for each year a substance was used was discussed in the context of memory cues such as developmental milestones, school grades, and relationships [20], [51]. For both self- report interviews, the following drug categories were assessed: ecstasy, marijuana, alcohol, sedatives (barbiturates, Valium, Xanax, Ativan, ketamine, GHB), stimulants (cocaine, crack cocaine, amphetamine, and methamphetamine), hallucinogens (PCP, LSD, peoyote, mushrooms), opioids (heroin, opium), and inhalants (paint, glue, household cleaners, nitrous oxide, gas). The unit of measure was the self-reported number of standard units of substances used (tablets for ecstasy; standard drinks for alcohol; joints for marijuana; grams for stimulants; number of hits for inhalants, hallucinogens, and opioids; and pills or hits for sedatives).

#### Family History of Substance Use Disorders (SUD)

Family history of SUD was obtained through a phone interview and positive status was given if any first or second-degree family member demonstrated at least one symptom of a known significant SUD (i.e., was diagnosed, received treatment, had relationships problems due to their use, had significant health problems due to their use).

#### Ecstasy Use Patterns

The Ecstasy Survey [20], [51] is a self-report instrument that measures ecstasy use patterns. Age of onset, how frequently ecstasy is simultaneously used with other drugs, maximum tablet consumption, subjective effects, and beliefs about the hazards of ecstasy use was measured utilizing a multiple-choice format.

#### Verbal Memory

The California Verbal Learning Test-2^nd^ Edition (CVLT-20 [67] was administered as part of a larger neuropsychological battery (see Medina et al. [20] for information about other cognitive domains). In this list-learning task, participants are read 20 words belonging to four semantic categories and asked to recall as many as possible across five learning trials. They are then read a 20-word distracter list and asked to recall the second list and then the first list. After this short delay, they are asked to recall the first list given cues about semantic categories. After a 20-minute delay, free recall and cued recall are measured. Following long-delayed recalls, recognition ability is measured. Nine CVLT-2 variables reflecting components of verbal memory were chosen: immediate recall (including trial 1 recall and recall for the interference list), short delayed recall (including free and cued recalls), long delayed recall (including free and cued recalls), retention (between short and long delayed), discriminability (examining correct inclusion of target items and exclusion of intrusion items for both recall measures), and recognition.

### Data Analysis

In order to examine potential confounding variables associated with ecstasy use, ANOVAs, chi-squares and Kruskal-Wallis tests were run on demographic and drug use variables to examine differences between ecstasy polydrug users and MJ users. Data was analyzed to determine whether the assumptions of multiple regressions were met. Analysis of histogram data revealed that past year ecstasy and other drug use were skewed to the right. As such, each drug use variable was log transformed to achieve normal distribution. However, the regression model was unable to support analysis with all log transformed drug use variables included. Further, prior literature has shown past year ecstasy use (raw) to be the most robust predictor of verbal memory, and the quota sampling technique utilized in recruitment and study inclusion yielded multiple bins of ecstasy exposure [20]. Additionally, ordinary least squares regression is very robust to non-normality in predictors so long as all dependent variables are normally distributed, which was confirmed upon review of histograms. Because of these issues, non-transformed past year drug use variables were included in the analyses below. Further, we conducted analysis of all DFBETAS for each case and regression coefficient. Based on Cohen and colleagues [68] a cut-off of score of.248 (N = 65) was utilized. All regression coefficients for every individual were well below this cut-off; in other words, none of the cases demonstrated significant influence for any of the predictors. Therefore, all participants were included in the regression analyses. Given the possible intercorrelations among the drug use variables, multicollinearity indices were closely examined through inspection of tolerance and VIF in regression models (e.g., relative reductions in tolerance and VIF >2.0). This resulted in the exclusion of past year hallucinogen and sedative usage (which were highly correlated with ecstasy use). All other drug use categories were included.

The primary analyses included a series of ordinary least squares multiple regressions that tested whether past year ecstasy exposure (number of tablets) or an interaction between ecstasy use and gender significantly predicted the CVLT-2 components of verbal memory after controlling for gender, ethnicity, WRAT 3 Reading [69] (which also reflects quality of education, see Manly [70]), family history of SUD, and frequency of other drug use (alcohol, marijuana, opioids, inhalants, and sedatives) within the entire sample of polydrug users. Because the CVLT-II is normed on age and gender only, ethnicity and reading ability were included in these analyses. In addition, the authors assessed regression findings within ecstasy users only, in order to compare results with that of the *full range* of ecstasy use among polydrug users (including non-use among MJ-using controls). Interpretations about statistical significance were made if *p*<.05.

Finally, it is important to note that the purpose of this examination was to assess several components of verbal memory, and this required multiple analyses. Because corrections for multiple comparisons, such as Bonferroni correction, tend to increase the likelihood of Type II error, the authors consulted with literature in this area [71]–[72]. Thus, the following results are presented without correction, and findings should be interpreted in this context.

## Results

### Demographic Information

As previously stated, data from ecstasy and marijuana users were combined in order to examine *ecstasy use as a continuous variable*. However, to test whether there were differences between ecstasy users and MJ users on demographic variables, ANOVAs and chi-squares were run. There were no significant differences between the ecstasy users and MJ users in length of education [*F* = 1.5(1, 64), *p*<.20], verbal ability [*F* = .03(1, 64), *p*<.80], or age [*F* = .35(1, 64), *p*<.60]. There was no difference in gender composition of groups [*x* ^2^(4) = .25, *p*<.61]. However, there was a significant difference in ethnic identification between groups, with a greater percentage of MJ users identifying as African American, which reflects the general ethnic distribution of the recruitment region [*x* ^2^(4) = 11.64, *p*<.02]. (See Table 1.)

**Table 1:** Breakdown of Ethnic Identification, Education, Reading Ability, and Age of Ecstasy and MJ Users.

Demographic Variable	Ecstasy Users (N = 48)		MJ Users (N = 17)		Chi-Square
Ethnic Identification	% Men	% Women	% Men	% Women	*p*<.02
Asian American	3.8	0	0	0	
African American	7.7	13.6	62.5	22.2	
Hispanic	0	0	12.5	0	
Caucasian	76.9	86.4	25	66.7	
Native American	0	0	0	0	
Other	11.5	0	0	11.1	
	%		%		Chi-Square
Gender (men)	54%		47%		*p*<.61
Family History (positive)	52%		53%		*p*<.95
	M	Range	M	Range	ANOVA
Education (years)	13	10–16	12.5	9–16	*p*<.22
Reading Ability	105.4	83–118	105.9	83–123	*p*<.86
Age	23.2	18–35	22.5	18–31	*p*<.56

### Drug Use Information

On average, ecstasy users had been abstinent from all drugs for 15 days (*M* = 15 days, *SD* = 17, range  = 7–117 days) and MJ users had been abstinent for one month (*M* = 31 days, *SD* = 89, range  = 7–378 days). Marijuana was the most recently used drug for the majority of participants. Ecstasy users had been abstinent from ecstasy for slightly more than five months on average (*M* = 161 days, *SD* = 128, range  = 11–491 days), and men and women did not differ in length of abstinence from ecstasy [*F* = .00(1, 46), *p* = .96]. Male ecstasy users reported *simultaneously* using alcohol with ecstasy significantly more than females [*F* = 5.98(1, 46), *p*<.02]. Table 2 provides a detailed description of the type and quantity of drug use during the past year by group and gender.

**Table 2:** Past Year Quantity of Drug Use (in Standard Units) According to Group and Gender.

	Ecstasy Users (N = 48)						MJ Users (N = 17)						
	*Males (N = 26)*			*Females (N = 22)*			*Males (N = 8)*			*Females (N = 9)*			Kruskal-Wallis
Drugs	M (N)	SD	Range	M (N)	SD	Range	M (N)	SD	Range	M (N)	SD	Range	p
Ecstasy	17 (26)	21	1–89	12 (22)	24	1–116	0	0	0	0	0	0	**.00**_a_
Alcohol	564 (25)	534	20–1836	244 (20)	204	14–662	296 (8)	297	2–906	356 (9)	537	8–1254	.13
Marijuana	603 (24)	910	1–3650	386 (20)	739	1–3185	306 (8)	189	7–608	256 (9)	262	5–728	.50
Opioids*	29 (7)	60	1–164	4 (3)	1	3–5	0	0	0	0	0	0	.12
Cocaine	21 (13)	31	1–110	2 (11)	3	1–8	54 (2)	70	4–103	0	0	0	**.05**_c_
Methamph.	5 (4)	9	1–18	10 (8)	23	1–67	0	0	0	0	0	0	**.03**_b_
Sedatives‡	21 (15)	43	2–150	14 (10)	17	1–51	0	0	0	2 (1)	0	2	**.01**_a_
LSD/PCP	5 (7)	6	1–18	2 (7)	2	1–5	0	0	0	0	0	0	.10
Mushrooms	10 (16)	22	1–91	3 (8)	4	1–12	0	0	0	0	0	0	**.00**_a_
Inhalants	15 (16)	30	1–104	10 (5)	5	5–18	0	0	0	0	0	0	.15

Mean quantities were calculated only for participants who reported using the specific drug at least one time in the past year.

*Includes heroin and opioids.

‡ Includes GHB, ketamine, barbiturates, ‘downers,’ Valium, Xanax, and Ativan. Methamph  =  methamphetamine. _a_ =  Ecstasy users > MJ users, _b_ =  Ecstasy females > all other groups, _c_ =  Ecstasy users > MJ females.

Kruskal-Wallis tests were conducted to evaluate differences in past year drug use between the four groups (male ecstasy users, female ecstasy users, male MJ users, and female MJ users). Follow-up tests (Holm's sequential Bonferroni approach) were conducted to evaluate pairwise differences controlling for Type I error. Ecstasy users (both male and female) had significantly greater past year ecstasy (*p* = .00), sedative (*p* = .01), and mushroom (*p* = .00) uses compared to male and female MJ users. Ecstasy users also had significantly more past year cocaine use compared to female MJ users (*p* = .05). Finally, female ecstasy users had significantly greater methamphetamine use compared to all other groups (*p* = .03). As stated above, the quantities of use for each of the drug categories were included as covariates in the regression models.

### Verbal Memory Functioning

For descriptive purposes, Table 3 provides the means, standard deviations, and ranges for the CVLT-2 z-scores. ANOVAs were run to examine the difference in mean z-score between ecstasy and marijuana users as a whole and by gender for each CVLT-2 variable. Qualitatively, with the exception of trial B, male and female ecstasy users performed worse than MJ users on each CVLT-2 variable (between.04 and.86 standard deviations lower).

**Table 3:** Mean, Standard Deviation, and Range of Verbal Memory Variables According to Group and Gender.

	Ecstasy Users (N = 48)						Marijuana Users (N = 17)						(E-MJ) z-score difference)	
	*Males (N = 26)*			*Females (N = 22)*			*Males (N = 8)*			*Females (N = 9)*				
CVLT-2 Variable	M	SD	Range	M	SD	Range	M	SD	Range	M	SD	Range	*Men*	*Women*
Trial 1	−.48	.90	3.00–1.50	−.40	1.29	−1.50–1.00	.25	1.31	−1.00–2.50	.25	1.31	−1.00–2.50	−.73	−.65
Trial B	−.94	.95	−3.00–.50	−.60	.22	−1.00–−.50	–1.00	.96	–3.00−0	−1.00	.96	−1.00–2.50	.06	.40
SDFR	−.40	1.05	−2.00–1.50	−.20	.84	−1.50–.50	0	.54	−.50–1.00	0	.53	−3.00–0	−.40	−.20
SDCR	−.52	.92	−2.50–1.00	−.50	1.23	−2.00–1.00	.19	.70	−1.00–1.00	.19	.70	−.50–1.00	−.72	−.69
LDFR	−.67	1.15	−3.00–1.00	−.60	1.08	−2.00–.50	.19	.84	−1.00–1.00	.19	.84	−1.00–1.00	−.86	−.79
LDCR	−.69	1.05	−3.00–1.00	−.60	1.34	−2.50–.50	.13	.84	−1.00–1.00	.13	.84	−1.00–1.00	−.82	−.73
LDFR vs. SDFR	−.27	.87	−2.50–2.00	−.40	.42	−1.00–0	.19	.53	−.50–1.00	.19	.53	−.50–1.00	−.46	−.59
Recall Disc.	−.50	.88	−2.00–1.50	−.50	.71	−1.50–0	.25	1.20	−1.50–2.00	.25	1.20	−1.50–2.00	−.75	−.75
Recog.	−.50	.91	−2.50–1.00	−.10	.55	−1.00–.50	−.06	.68	−1.00–1.00	−.06	.68	−1.00–1.00	−.44	−.04

Z-scores are reported except retention (positive scores indicate poorer retention). (E-MJ) indicates the difference in mean scores between the ecstasy (E) and MJ users for all components of verbal memory except retention. Abbreviations: SDFR  =  Short Delay Free Recall, SDCR  =  Short Delay Cued Recall, LDFR  =  Long Delay Free Recall, LDCR  =  Long Delay Cued Recall, LDFR vs. SDFR =  Long Delay Free Recall vs. Short Delay Free Recall, Recall Disc.  =  Recall discriminability, Recog.  =  Total recognition.

### Bivariate Relationships

See Table 4 for the significant bivariate relationships (Pearson product moment correlations) between the verbal memory variables and ecstasy and other drug usage variables (past year). Increased past year ecstasy use was significantly associated with poorer performance on all of the verbal memory variables except trial B performance (interference list). As previously stated, past year ecstasy use was used in the analyses because it was the most robust predictor of cognitive functioning in previous studies [20].

**Table 4:** Simple Relationships between Components of Verbal Memory and Drug Usage Variables (Past Year).

	Trial 1	Trial B	SDFR	SDCR	LDFR	LDCR	LDFR vs. SDFR	Recall Disc.	Recog.
Ecstasy	−.26*	−.19	−.26*	−.39*	−.40*	−.33**	−.28*	−.37**	−.32**
Alcohol	.16	.10	−.12	−.17	.01	−.07	.16	−.11	−.25*
Marijuana	−.23	−.05	.05	−.22	.07	−.05	.04	.01	−.28*
Stimulants	.08	−.05	−.17	.05	.05	.07	.36*	−.10	−.16
Opioids	.06	−.21	−.14	−.17	−.13	−.06	−.08	−.12	−.07
Sedatives	0.00	−.25*	−.11	−.28*	−.16	−.16	−.10	−.15	−.11
Hallucinogens	−.13	−.27*	−.22	−.34**	−.18	−.25*	.01	−.22	−.24
Inhalants	.08	.12	−.20	−.01	−.04	.01	.20	.01	−.01

Correlations are Pearson Product Moment Correlations. Abbreviations: SDFR  =  Short Delay Free Recall, SDCR  =  Short Delay Cued Recall, LDFR  =  Long Delay Free Recall, LDCR  =  Long Delay Cued Recall, LDFR vs. SDFR  =  Long Delay Free Recall vs. Short Delay Free Recall, Recall Disc.  =  Recall discriminability, Recog.  =  Total recognition.

*p<.05.

**p<.01.

### Multivariate Relationships

#### Past Year Ecstasy Use & Verbal Memory: Full range of ecstasy use

**Figure 1:**
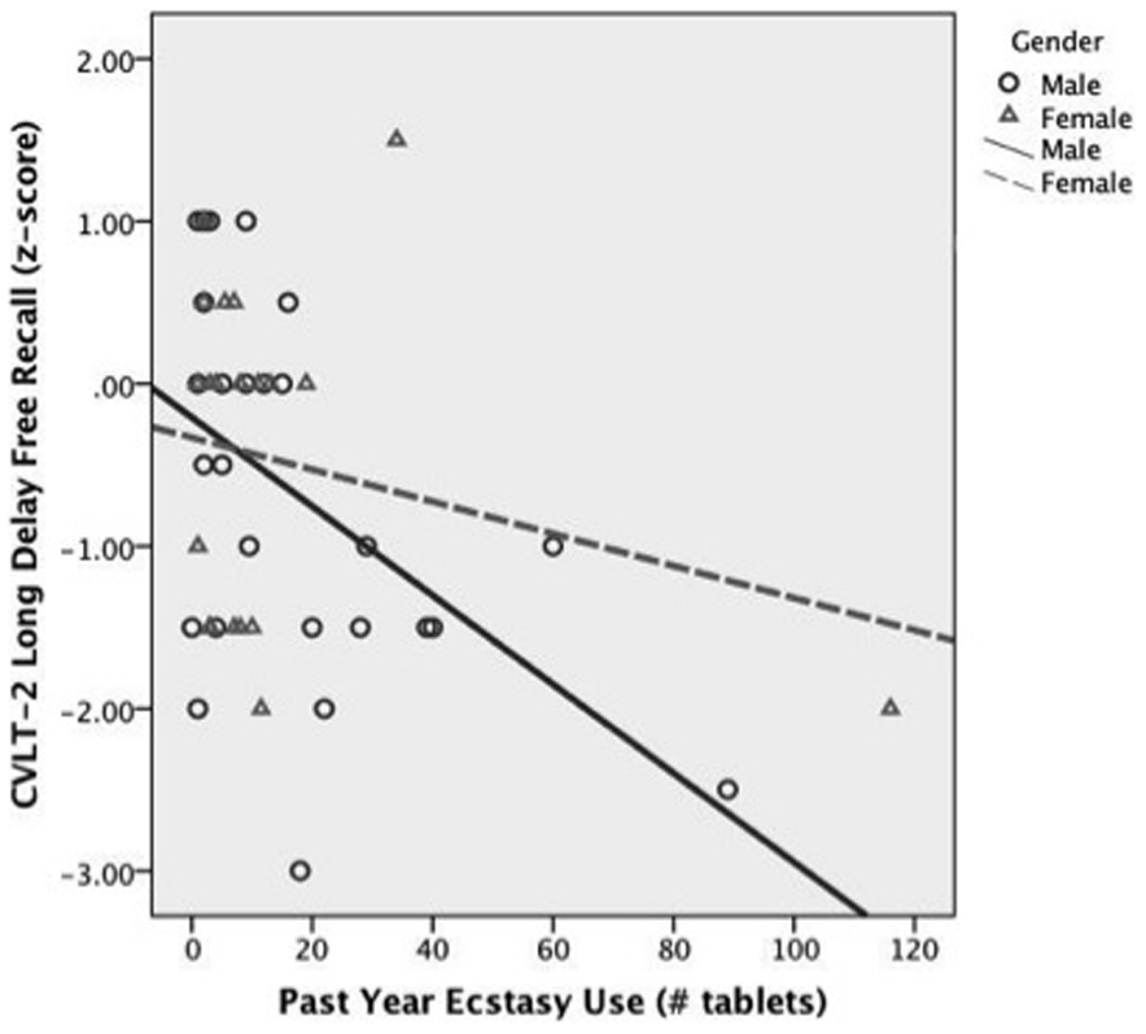
Bivariate Relationship between CVLT-2 Retention z-score and Past Year Ecstasy Use According to Gender.

After statistically controlling for past year consumption of other drugs and potential confounding demographic variables (gender, ethnicity, reading ability, family history of SUD), increased past year ecstasy use was associated with poorer short delayed free [*beta*  = −.31, *p*<.02] and cued recall [*beta*  = −.33, *p*<.02], long delay free [*beta*  = −.43, *p*<.001] and cued recall [*beta*  = −.28, *p*<.04], retention between long and short delays [*beta*  = −.26, *p*<.04], recall discriminability [*beta*  = −.34, *p*<.01]. Past year ecstasy use did not predict trial 1 recall [*beta*  = −.17, *p*<.24] or trial B recall [*beta*  = −.23, *p*<.14]. Past year ecstasy use interacted with gender in significantly predicting retention between long and short delays [*beta*  = .35, *p*<.003]; male ecstasy users demonstrated a more robust relationship between increased past year ecstasy use and poorer verbal memory performance compared to female users (see Fig. 1).

#### Past Year Ecstasy Use & Verbal Memory: Ecstasy users only

Among ecstasy users only, all significant predictions of past year ecstasy use were consistent with those reported in the full sample, with the exception of long delay cued recall and retention [*beta*  = −.01, *p* = .14; *beta*  = −.01, *p* = .46, respectively]. As before, the significant interaction between increased past year ecstasy use and gender predicted retention in the same direction [*beta*  = .02, *p* = .009].

#### Comorbid Drug Use & Verbal Memory

Greater alcohol use was associated with poorer short delay cued recall [*beta*  = −.26, *p*<.04; not significant in ecstasy only analyses] and recognition ability [*beta*  = −.28, *p*<.04; same in ecstasy only analyses]. Increased inhalant use was associated with poorer short delay free recall [*beta*  = −.26, *p*<.05]; in ecstasy only analyses increased inhalant use was still associated with poorer short delay free recall as well as better retention [*beta*  = .01, *p*<05]. Increased stimulant use was associated with poorer short delay free recall [*beta*  = −.27, *p*<.05]; in ecstasy only analyses increased stimulant use was still associated with poorer short delay free recall as well as better retention [*beta*  = .01, *p*<01].

#### Length of abstinence from ecstasy

Pearson correlations were run in the male and female ecstasy users to examine the relationship between length of abstinence from ecstasy and verbal memory functioning. As shown in Table 5, the female users (*n* = 26) generally demonstrated improved verbal memory with increased ecstasy abstinence, with significant positive relationships between ecstasy abstinence and short delayed cued recall and recognition ability and moderate relationships with immediate recall, short delay free recall, long delay free and cued recall and recall discriminability. In contrast, no significant relationships were observed in the male ecstasy users (*n* = 26). Fisher z calculations revealed that females had significantly more robust relationships between ecstasy abstinence and short delay cued recall (*p*<.10) and recognition ability compared to men (*p*<.05).

**Table 5:** Simple Relationships between Components of Verbal Memory and Ecstasy Abstinence by Gender.

	Ecstasy Abstinence		
	Females (*n* = 22)	Males (*n* = 26)	Fisher's z
Trial 1	.36*	.03	1.12
Trial B	.23	.24	−.03
SDFR	.41*	.27	.51
SDCR	.49**	.04	1.6*
LDFR	.36*	.09	.92
LDCR	.41*	.08	1.15
LDFR vs. SDFR	−.03	−.20	0.56
Recall Disc.	.40*	.26	0.51
Recog.	.58**	−.04	2.27**

Correlations are Pearson Product Moment Correlations. Fisher's z statistics were calculated to determine whether correlations were significantly different between male and female ecstasy users. Abbreviations: SDFR  =  Short Delay Free Recall, SDCR  =  Short Delay Cued Recall, LDFR  =  Long Delay Free Recall, LDCR  =  Long Delay Cued Recall, LDFR vs. SDFR  =  Long Delay Free Recall vs. Short Delay Free Recall, Recall Disc.  =  Recall discriminability, Recog.  =  Total recognition.

*p<.10.

**p<.05.

## Discussion

This study examined whether ecstasy exposure and gender interact in predicting component processes of verbal memory as measured by a list-learning task in 65 young adult ecstasy polydrug users with a full range of ecstasy exposure (from no use to heavy) after controlling for comorbid drug use and important demographic variables including family history of SUD. Increased past year ecstasy use significantly predicted all but 3 of the 9 CVLT-2 verbal memory components in a dose-dependent fashion after controlling for polydrug use and potentially confounding variables. Specifically, this included variables measuring short and long delayed free and cued recall and retention of previously learned material. When the model was examined with only individuals with ecstasy use, increased use predicted 4 of 9 variables. In addition, male ecstasy users had a particularly robust relationship between past year ecstasy exposure and retention compared to female users in both models. Increased alcohol use was independently associated with poorer short delayed cued recall and recognition; increased inhalant and stimulant use were independently associated with poorer short delayed free recall. Finally, increased abstinence from ecstasy was significantly associated with improved verbal memory functioning in the females, especially in short delay cued recall and recognition ability.

The primary findings of this study suggest that past year ecstasy consumption negatively impacts several components of verbal memory (retrieval, retention, and recall discrimination) for both men and women. In fact, ecstasy users performed, on average,.60 standard deviations below published norms and.47 standard deviations below the MJ polydrug controls. These are clinically significant differences for several reasons: ecstasy polydrug users were otherwise healthy young adults who had no independent comorbid Axis I disorders (besides possible ADHD and conduct disorder), no neurological injuries, and had been abstinent from ecstasy for an average of five months. With few exceptions [28]–[30], these results are consistent with much of the literature regarding ecstasy's effect on immediate and delayed verbal memory [19], [38]–[43]. However, few studies have broken down verbal memory into components aside from immediate versus delayed recall. Quednow et al. [41] examined the relationship between ecstasy exposure and components of verbal memory (measured by the RAVLT) in a sample of male ecstasy polydrug users, MJ users, and normal controls. Although they found reduced immediate and delayed recall, they reported spared cued recall and recognition ability. This discrepancy may have been due to differences in extent of ecstasy exposure, polydrug use patterns and statistical power. For example, Halpern and colleagues [29] reported intact verbal memory functioning in a sample of ecstasy users who demonstrated less lifetime exposure, but most notably also had substantially less lifetime alcohol, marijuana and other illicit drug use and all users were abstinent from marijuana. Although the current study statistically controlled for comorbid drug use (with the exception of hallucinogens and sedatives), our ecstasy users were generally heavier drug users and may have also engaged in more simultaneous drug use, although Halpern and colleagues did not report the latter. Therefore, the current findings provide further evidence that increased ecstasy use, *within the context of polydrug use*, is associated with verbal memory deficits.

Potential combined drug use may have also contributed to our gender findings. The current study found that male ecstasy users were more likely to take ecstasy *simultaneously* with alcohol compared to female users (*p*<.02), who were more likely to use ecstasy alone with no other substances. While there is some research supporting possible neuroprotective effects of simultaneous substance use [73], at least one study found that binge ethanol administration prior to MDMA exposure *increased* the neurotoxic effects of MDMA in the hippocampus in rats, regardless of an increase in body temperature (alcohol decreases whereas ecstasy is associated with increased body temperature) [74]. Additional research in humans is needed to examine the effects of simultaneous use of ecstasy and other substances, particularly alcohol and marijuana, on neurocognitive functioning.

Results suggest that male ecstasy users exhibit a more robust relationship between ecstasy exposure and poorer performance on retention, with a trend towards poorer retrieval of information, especially following a long delay. This result is consistent with Bolla et al. [61], as male users performed more poorly than female users in a dose-dependent relationship on delayed recall and measures of retention. Given these findings, ecstasy use may reduce neurogenesis in the hippocampus through its impact on the serotonin system, most especially in males [21]–[22], [75]–[82]. This is in contrast with Reneman et al. [60], who noted increased sensitivity of the serotonin system in female ecstasy users as reflected by reduced number serotonin transporters. However, females demonstrated an increased number of transporters following one year of abstinence. Perhaps females are more susceptible to serotonergic neurotoxicity during use, but demonstrate an increased ability to recover following abstinence. Indeed, our current data support this hypothesis, at least within the constraints of a cross-sectional design. Compared to the males, female ecstasy users in the current study demonstrated a significantly more robust relationship between increased ecstasy abstinence and improved short delay cued recall and recognition ability. It is worth noting that such differential gender findings may be related to the composition of the each gender group. Table 2 lists past year quantity of drug use only for those individuals who endorsed use. In the ecstasy group, men demonstrated increased polydrug use compared to women, especially for mushroom and inhalant use; conversely, female ecstasy users reported greater meth use than male ecstasy users. While these differences were not statistically significant, they do suggest relatively increased polydrug use patterns among males and may help explain our gender differences findings. Future longitudinal research will be necessary to confirm the differential trajectories in recovery between male and female ecstasy users, with the consideration and analysis of patterns of polydrug use behavior.

Potential limitations of the current study need to be considered. First, there is no control group for comparison to the drug-using group. However, we specifically modeled the groups to assess for the unique contribution of ecstasy dose within typical polydrug users (with and without ecstasy use). We believe that these findings are still clinically and statistically meaningful given the dose-dependent pattern of deficits as well as the poorer performance relative to published norms. In addition, there may be additional limitations related to the variation in ecstasy use among the entire sample. As mentioned in the data analysis section, we chose to include raw past year drug use and examine the full range of past year ecstasy use (including none) in the regression as ordinary least squares regression is very robust to non-normality in predictors when DVs are normally distributed. However, there is evidence that the strength of some relationships, specifically 2 of the components that were significant in the full model, were no longer significant when examined in the ecstasy only model. This may demonstrate that results are optimally robust when the full range of ecstasy use (including none); reduced power when removing 35% of the sample should also be considered. Of note, an examination of scatterplots suggested two ecstasy users (one male with 89 past year uses and one female with 116 past year uses) were outliers in their past year ecstasy use. Although these two may appear to be outliers in their ecstasy use, they were purposely sampled for in order to examine dose-dependent relationships; hence, we were more concerned about whether or not these cases demonstrated *undue influence* on each ecstasy and ecstasy-by-gender regression coefficients when predicting the verbal memory variables. Analyses revealed that all DFBETAS were <.248, the recommended cut off given our sample size [68]. Therefore, we did not exclude any participants from the analysis based on their ecstasy exposure. Second, as mentioned earlier, ecstasy users were more likely to use cocaine, sedatives, methamphetamine and mushrooms than MJ users. Although polydrug use was controlled for in analyses, issues related to multicollinearity required inability to control for hallucinogen and sedative use in regression analyses, and our findings must be interpreted within the context of polydrug use. Indeed, Daumann and colleagues [83] noted brain activation differences between pure and polydrug ecstasy users during a working memory task, and authors suggested that polydrug use in the sample may have modified the findings associated with ecstasy use alone. As mentioned earlier, the present findings did not undergo correction for multiple comparisons due to the nature of the study of components of verbal memory as well as the tendency for increased Type II errors in such conservative corrections (e.g., Bonferroni). However, the authors noted that some of our findings survived a less conservative alpha criterion of 1%, for the prediction of ecstasy use on long delay free (*p*<.001), recall discriminability (*p*<.01), and the interaction with gender on retention (*p*<.003; for the analysis with the full range of ecstasy use). In addition, the amount of MDMA in the ecstasy tablets consumed by participants was unknown, and thus it is important to note that the dose-dependent effects reported in this study were related to what participants considered “ecstasy,” which typically contains primarily MDMA but may also contain other substances such as MDA (methylenedioxyamphetamine), ephedrine, caffeine, DXM (dextromethorphan), ketamine, PMA (paramethoxyamphetamine), cocaine and methamphetamine.

Another limitation to this study was that participants were not required to submit to a drug toxicology test to assess for current intoxication and to corroborate self-reported recency of drug use. Unfortunately, one testing session would not have captured the length of abstinence from ecstasy required (7 days), as MDMA is typically out of the user's system within 2–4 days. On the other hand, there is a lengthy detection window for THC captured by traditional urinalysis (from several days to several weeks), meaning that it would be likely that regular-using individuals reporting at least 7 days of abstinence would still have THC in their systems. As such, at least 2 toxicology sessions to confirm abstinence would have been ideal. Thus, use within the 7-day abstinence period cannot be ruled out and may have contributed to present findings. However, every attempt was made to maximize the reliability of users' self-reports. This included expressed confidentiality, privacy, and double assessment of length of abstinence. The *Time Line Follow-Back* technique was employed, which has demonstrated high re-test reliability and convergent and discriminant validity compared to other established measures, high agreement with collateral informants and patient's urine assays [66]. In fact, a recent meta-analysis reported high agreement (nearly 90%) between *Time Line Follow-Back* self-report and biological assays across 29 studies [84]. Future studies should require at least 2 toxicology screenings, with advanced testing assessing THC metabolites, to confirm self-reported abstinence as well as sobriety on session day. In addition, we did not measure the proportion of ecstasy ingested relative to body weight; it is possible women may have had greater exposure because of lower average body weight and increased body fat percentage, although this hypothesis do not explain the *poorer* performance in the men given similar ecstasy use across genders. Risk factors associated with drug use, including family history of SUD, conduct disorder and attention deficit hyperactivity disorder (ADHD) are themselves related to cognitive function and diagnoses can differ by gender [85]–[86]. It is notable that the current study did exclude for independent psychiatric mood, anxiety, and psychotic disorders, and we statistically controlled for family history of SUD, which was not a significant predictor in any of the models; still, individuals with conduct disorder and ADHD were not excluded. Therefore, further research is necessary to disentangle the potential effects of these premorbid factors.

In conclusion, we found that increased ecstasy exposure was associated with nearly every component of verbal memory assessed, including proactive interference, delayed memory, retention of previously learned information, reduced discrimination ability, and poorer recognition. Such deficits were notable in a sample of physically healthy young adults without a history of psychiatric or neurological disorders, even after controlling for comorbid drug use and important demographic variables (including family history of SUD). In addition, male ecstasy users were more vulnerable to deficits in retention following a long delay compared to female users. Results highlight the considerable deficits in verbal memory associated with ecstasy consumption among both men and women who engage in polydrug use, even among recreational users (1–2 tablets per month on average). Future studies are needed to determine underlying etiology of this gender difference in verbal memory long delay retention.

Given the observed deficits in verbal memory, an examination of possible recovery of function with greater prolonged abstinence from ecstasy [87] and treatment programs aimed at improving cognitive functioning (e.g., exercise, cognitive rehabilitation) are needed. Studies aimed at determining individual differences in neurocognitive consequences of ecstasy use in young adults are also needed. Potential targets include different patterns of drug use- such as simultaneous ecstasy and binge drinking patterns [74], body mass index [88], extent of physical activity [89], and variability in genes regulating growth factors or serotonin functioning such as *BDNF* [90]–[92] or *5-HTTLPR* [93].
